# Identification of a Novel Saxitoxin Analogue, 12β-Deoxygonyautoxin 3, in the Cyanobacterium, *Anabaena circinalis* (TA04)

**DOI:** 10.3390/toxins11090539

**Published:** 2019-09-16

**Authors:** Takashi Minowa, Yuko Cho, Yasukatsu Oshima, Keiichi Konoki, Mari Yotsu-Yamashita

**Affiliations:** 1 Graduate School of Agricultural Science, Tohoku University, 468-1 Aramaki-Aza-Aoba, Aoba-ku, Sendai 980–8572, Japan; 2 Graduate School of Life Sciences, Tohoku University, 2-1-1 Katahira, Aoba-ku, Sendai 980–8577, Japan

**Keywords:** saxitoxin, gonyautoxin, biosynthesis, *Anabaena circinalis*, paralytic shellfish toxins

## Abstract

Saxitoxin (STX) and its analogues, the potent voltage-gated sodium channel blockers, are biosynthesized by freshwater cyanobacteria and marine dinoflagellates. We previously identified several biosynthetic intermediates in the extract of the cyanobacterium, *Anabaena circinalis* (TA04), that are primarily produced during the early and middle stages in the biosynthetic pathway to produce STX. These findings allowed us to propose a putative biosynthetic pathway responsible for STX production based on the structures of these intermediates. In the present study, we identified 12β-deoxygonyautoxin 3 (12β-deoxyGTX3), a novel STX analogue produced by *A. circinalis* (TA04), by comparing the retention time and MS/MS fragmentation pattern with those of synthetic standards using LC–MS. The presence of this compound in *A. circinalis* (TA04) is consistent with stereoselective enzymatic oxidations at C11 and C12, and 11-*O*-sulfation, during the late stage of STX biosynthesis, as proposed in previous studies.

## 1. Introduction

**Figure 1:**
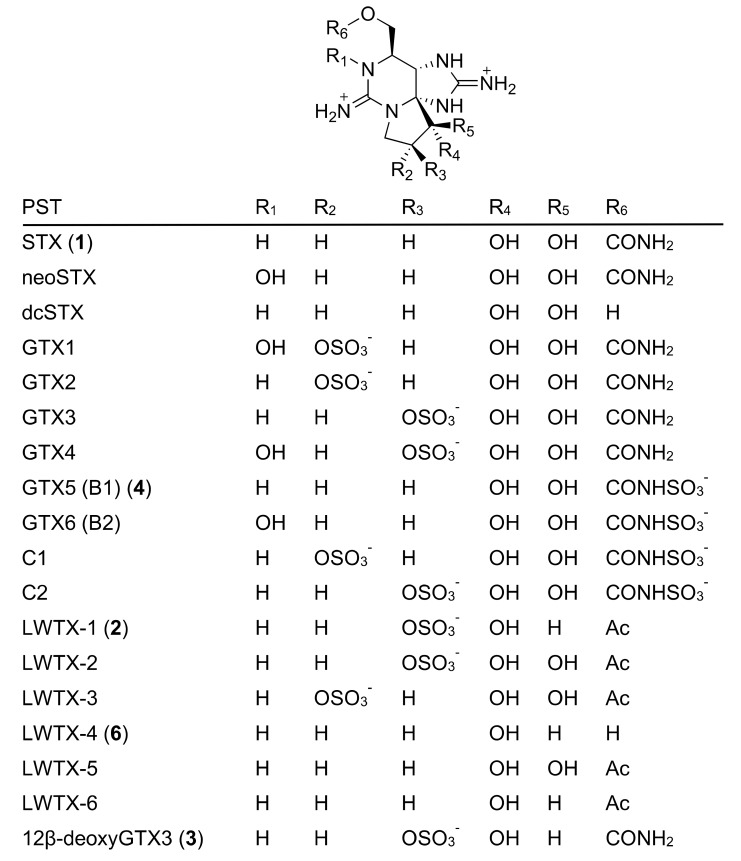
Structures of saxitoxin (STX) (**1**), a novel natural STX analogue 12β-deoxyGTX3 (**3**), and some other natural paralytic shellfish toxins (PST).

Saxitoxin (STX (**1**), Figure 1) and its analogues, the potent voltage-gated sodium channel blockers [1], are known as paralytic shellfish toxins (PST) [2,3]. Nearly 60 natural analogues of STX have been reported [4]. Several species of freshwater cyanobacteria and marine dinoflagellates have been identified to produce PST [2,3,4,5]. The biosynthetic pathway responsible for the synthesis of STX was first proposed by Shimizu et al. [6] by conducting feeding experiments using stable isotope-labeled acetic acid and amino acids as essential substrates with PST producing cyanobacteria and dinoflagellates. Next, Neilan’s group discovered putative STX biosynthesis gene clusters (*sxt*) in the cyanobacterium, *Clyndrospermopsis raciborskii* T3 [7]. The majority of the core set of genes (*sxtA-sxtI, sxtP-sxtR, sxtS*, and *sxtU*) were commonly identified in PST producing cyanobacteria [4]. Homologous genes to some of these genes were also found in PST producing dinoflagellate strains [8,9]. Concerning the *O*-sulfonation and *N*-sulfonation enzymes, Sako et al. [10] purified and characterized a sulfotransferase specific to *N*-21 of STX and gonyautoxin (GTX) 2/3, and Yoshida et al. [11] characterized a sulfotransferase specific to *O*-22 of 11-hydroxysaxitoxin; both enzymes were detected in the toxic marine dinoflagellate, *Gymnodium catenatum*. We previously identified biosynthetic intermediates of STX, namely, Int-A’, Int-C’2, Int-E’, 11-hydroxy Int-C’2, and a shunt compound, Cyclic-C’, in the PST producing freshwater cyanobacterium, *Anabaena circinalis* (TA04), and the toxic marine dinoflagellate, *Alexandrium tamarense* (Axat-2), using synthetic standards and LC–MS, along with feeding experiments. The above findings allowed us to propose a putative biosynthetic pathway responsible for STX production based on the structures of these intermediates [12,13,14,15]. Recently, Narayan’s group demonstrated the functions of some enzymes encoded in cyanobacterial PST biosynthetic gene clusters and showed a polyketide-like synthase, SxtA [16], and the enzymes that catalyze C–H hydroxylation are SxtT, SxtH, and GxtA [17]. In addition, SxtSUL and SxtN from *C. raciborskii* T3 were demonstrated to act as an *O*-sulfotransferase and *N*-sulfotransferase, respectively [18].

Freshwater cyanobacteria are also known as useful sources of various PST analogues. Six STX analogues, namely, LWTX-1 (**2**, Figure 1) and LWTX-2–6, were isolated from the freshwater cyanobacterium, *Lyngbya wollei,* and their corresponding chemical structures were determined by NMR spectroscopic analysis by Onodera et al. [19] Five of these analogues contain an *O*-acethyl moiety at C13 instead of the *O*-carbamoyl moiety in STX, and three of them harbor an α-hydroxyl group instead of a hydrated ketone at C12 in STX. The presence of these analogues implies a broad range of metabolic reactions that occur during the late stage of PST biosynthesis in the cyanobacteria. Hudon et al. [20] examined spatial and temporal variations of LWTX-1 (**2**) in *L. wollei* mats in the St. Lawrence River (Quebec, Canada) to monitor this cyanobacterium in the environment. Recently, D’Agostino et al. [21] reevaluated the PST profiles of six cyanobacteria using LC–MS and detected a total of 35 different PST variants (some of them are shown in Figure 1).

In the present study, we screened for novel PST-related compounds to analyze PST biosynthesis based on the structures of the analogues. Using LC–MS, we identified a novel PST analogue in *A. circinalis* (TA04) extract, and the structure was determined to be 12β-deoxygonyautoxin 3 (12β-deoxyGTX3) (**3**) by comparison with synthetic standards. The putative biosynthetic route to this compound was additionally predicted based on previously reported biosynthetic reactions.

## 2. Results

### 2.1. Screening for Novel PST in A. circinalis (TA04)

**Figure 2:**
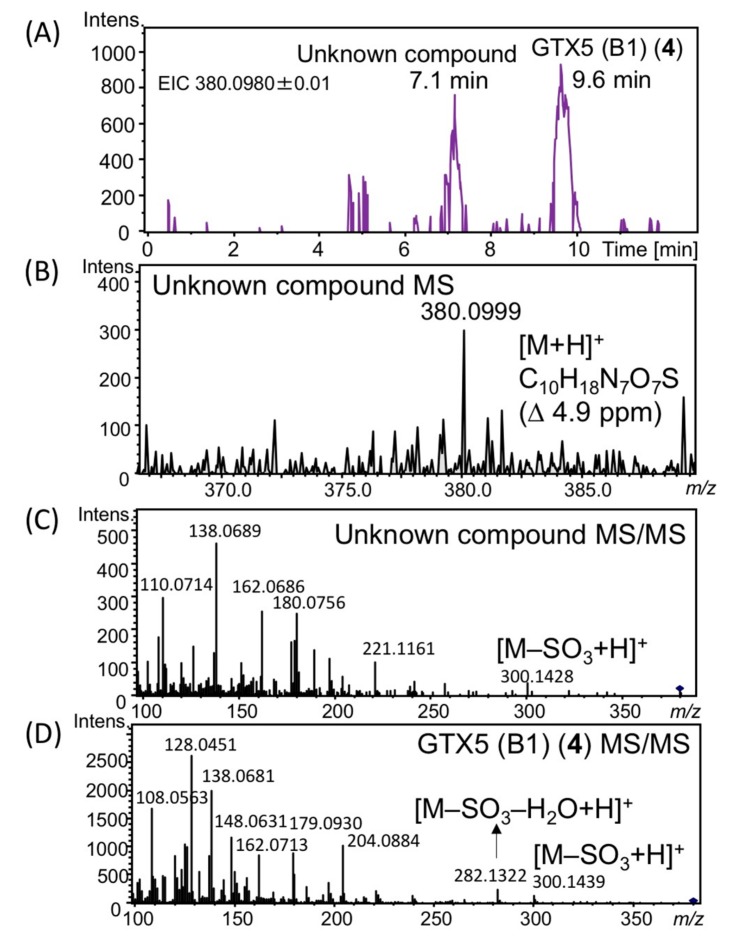
Detection of an unknown PST analogue in *A. circinalis* (TA04). (**A**) HR–LC–MS EIC detected at *m*/*z* 380.0980 ± 0.01; (**B**) MS spectrum of the unknown PST analogue shown at 7.1 min; (**C**) MS/MS spectrum of the semipurified unknown PST analogue shown at 7.1 min; (**D**) MS/MS spectrum of the authentic GTX5 (B1) (**4**). Chromatographic condition; column: TSKgel Amide-80 (2.0 × 150 mm, 5 µm), solvent: 2 mM HCOONH_4_ in CH_3_CN-H_2_O (62:38, *v*/*v*), flow rate: 0.2 mL min^−1^.

Screening for novel PST in *A. circinalis* (TA04) resulted in detecting an unknown peak at 7.1 min on the extracted ion chromatogram (EIC) at *m*/*z* 380.0980 ± 0.01, whereas the peak of the known PST-analogue, GTX5 (B1) (**4**) [22,23], was detected at 9.6 min (Figure 2A). The high-resolution (HR) MS ([M+H]^+^) of this unknown compound was *m*/*z* 380.0999 (C_10_H_18_N_7_O_7_S, ∆ 4.9 ppm, Figure 2B). The MS/MS spectrum (Figure 2C) showed a desulfate ion [M–SO_3_+H] ^+^ at *m*/*z* 300.1428, suggesting the presence of an SO_3_H moiety in this molecule, similar to GTX5 (B1) (**4**) (Figure 2D). However, this unknown compound did not show a dehydrated ion [M–SO_3_–H_2_O+H]^+^ (C_10_H_16_H_7_O_3_^+^) *m*/*z* 282.1309, whereas STX analogues that contain a hydrate ketone at C12 commonly show a dehydrated product ion in their MS/MS spectra [24,25]. For example, the MS/MS spectrum of GTX5 (B1) (**4**) showed the ion corresponding to [M–SO_3_–H_2_O+H]^+^ (C_10_H_16_H_7_O_3_^+^) at *m*/*z* 282.1309 (Figure 2D). Based on the above results, this novel STX analogue is a 12-deoxy type analogue and is most likely to be 12β-deoxyGTX2 or 12β-deoxyGTX3 (**3**), because 12α-deoxy analogues have not been identified in natural sources. Therefore, we prepared 12β-deoxyGTX2 and 12β-deoxyGTX3 (**3**), along with the mixture of 12α-deoxyGTX2/3, by chemical derivatization from C1/C2 to compare the chemical data.

### 2.2. Preparation and Spectroscopic Identification of 12β-deoxyGTX2 (**5**) and 12β-deoxyGTX3 (**3**)

**Figure 3:**
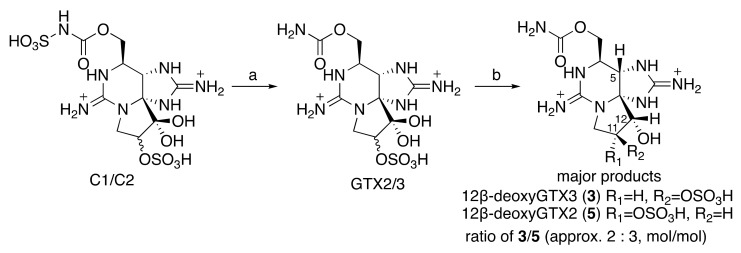
Preparation of compounds **3** and **5** from C1/C2. (**a**) 0.13 M HCl aqueous 100 °C, 15 min. [26] (**b**) NaBH_4_ water 0 °C, 30 min. [27].

12β-deoxyGTX2 (**5**) and 12β-deoxyGTX3 (**3**) were chemically derived from C1/C2, the predominant PST found in *A. circinalis* (TA04) (Figure 3). First, C1/C2 purified from *A. circinalis* (TA04) culture was hydrolyzed (*N*-desulfated) to GTX2/3 following a previously reported method [26]. Next, the hydrated ketone at C12 in GTX2/3 was reduced with NaBH_4_ in water at 0 °C to produce a mixture of 12α-deoxyGTX2/3 and 12β-deoxyGTX2/3 (the ratio of 12α- GTX2/3 and 12β-GTX2/3 was approximately 1:50, mol/mol, by HILIC-LC/MS), as reported by Koehn et al. [27] Thereafter, from the reaction mixture, 12β-deoxyGTX2 (**5**) and 12β-deoxyGTX3 (**3**) were isolated by reverse-phase column chromatography (**3** was eluted earlier than **5**, Figure S1). The estimated ratio between **3** to **5** in the reaction mixture was approximately 2:3 (mol/mol) by LC/MS. The yields of the purified **3** and **5** from C1/C2 were approximately 7% and 10% (mol/mol), respectively.

**Figure 4:**
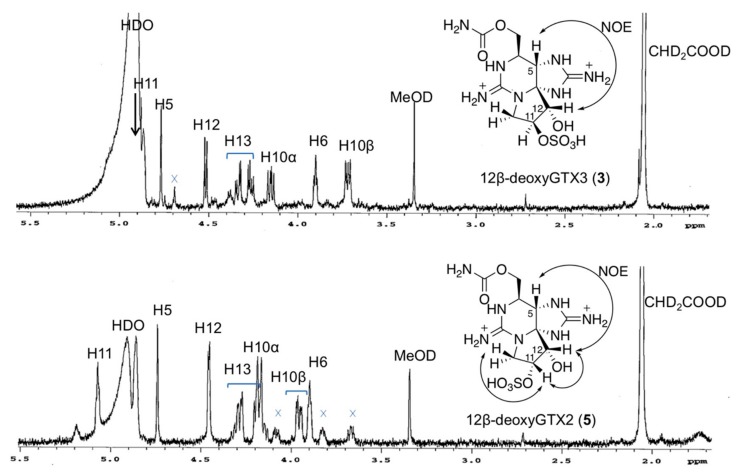
The ^1^H NMR spectra of synthetic 12β-deoxyGTX3 (**3**) and 12β-deoxyGTX2 (**5**) and observed key NOEs in these compounds. 600 MHz, solvent: CD_3_COOD-D_2_O (4:96, *v*/*v*). The HDO signal was suppressed. The signal of CHD_2_COOD (2.06 ppm) was used as an internal reference.

Purified compounds **3** (HRMS [M + H]^+^ *m*/*z* 380.0968 C_10_H_18_N_7_O_7_S, ∆ 3.9 ppm, Figure S2) and **5** (HRMS *m*/*z* 380.0987 C_10_H_18_N_7_O_7_S, ∆ 1.1 ppm, Figure S3) were analyzed by ^1^H NMR spectroscopy to determine the stereochemistry at C11 and C12 (Figure 4). The ^1^H NMR signals were assigned based on COSY and TOCSY correlations (Figures S4–S7), and comparison of ^1^H NMR data with those of LWTX-1 (**2**) (Table 1). The α-orientation of 12-OH in **3** and **5** was confirmed by the observed NOEs between H5 and H12 on their NOESY1D spectra (Figure 4). The stereochemistry at C11 of **3** and **5** was determined by comparing the ^1^H NMR data with that of LWTX-1 (**2**) [19] (Table 1). The chemical shifts of the ^1^H NMR signals of compound **3** were close to those of **2**; the differences between the proton chemical shifts were ≤0.05 ppm, whereas the chemical shift differences between **5** and **2** for H10β, H11, and H13a were 0.19, 0.17, and 0.33 ppm, respectively, which were higher than those of **3**. These data suggested that the stereochemistry at C11 of **3** is the same as that of **2**, whereas the stereochemistry at C11 of **5** is the opposite from that of **2**. Furthermore, NOEs between H11 and H12, and H11 and H10β were observed in **5**, but not in **2** (Figure 4). In addition, the ^3^*J*_H11/H12_ value of **3** (7.0 Hz) (Table 1) was the same as that that of **2** (7.0 Hz), whereas that of **5** (4.0 Hz) was smaller than that of **2**. Taken together, these results support the β-orientation of C11-OSO_3_H in **3** and α-orientation of that in **5**.

### 2.3. Identification of 12β-deoxyGTX3 (**3**) in A. circinalis (TA04)

**Figure 5:**
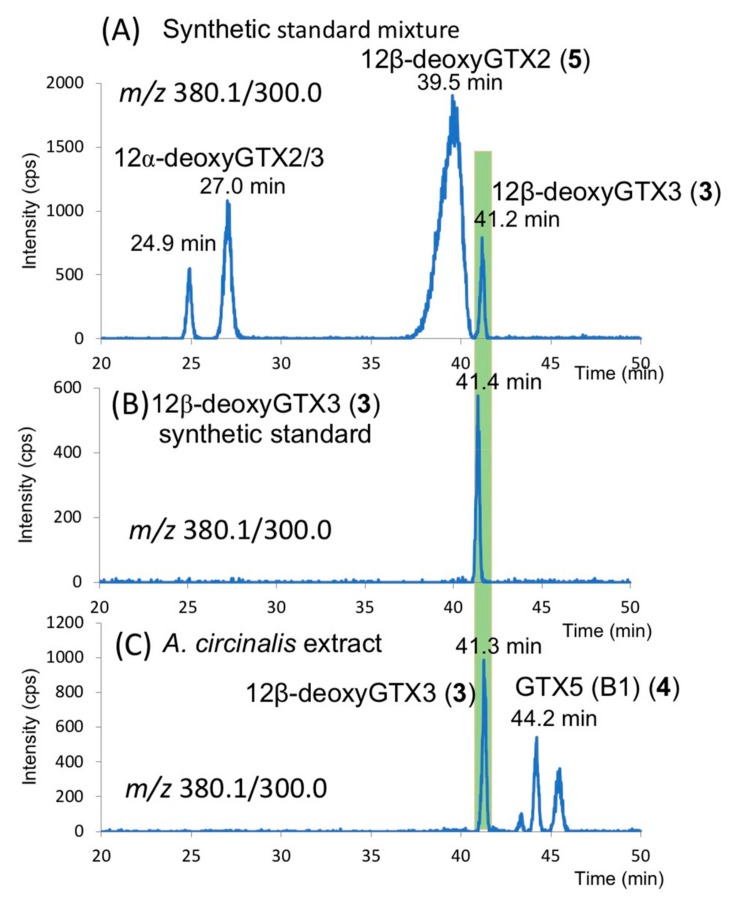
The LC–MS/MS multiple reaction monitoring (MRM) chromatograms of (**A**) synthetic standard mixture of 12α-deoxyGTX2/3 and 12β-deoxyGTX2/3, (**B**) synthetic standard of 12β-deoxyGTX3 (**3**), (**C**) *A. circinalis* (TA04) extract. LC was performed under the column switching condition [28].

**Figure 6:**
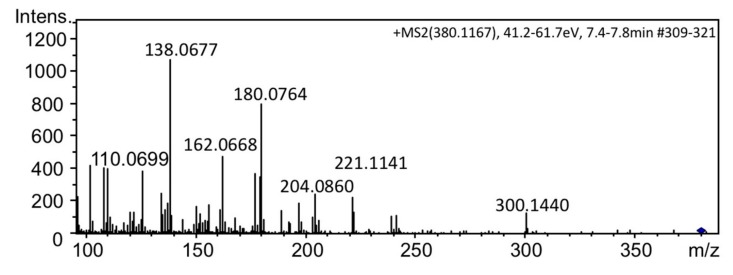
The MS/MS spectrum of synthetic 12β-deoxyGTX3 (**3**).

The extracts of the cells of *A. circinalis* (TA04) were treated with activated charcoal (for chromatography, 63–300 µm >40%), and then subjected to LC–MS analysis in multiple reaction monitoring (MRM) mode under the column switching condition [28] to compare the retention times of the unknown PST with that of the synthetic 12α-deoxyGTX2/3 and 12β-deoxyGTX2/3 (**5**, **3**) (Figure 5). The peak at 41.3 min observed in the extract of *A. circinalis* (TA04) (Figure 5C) was consistent with the retention time of the synthetic 12β-deoxyGTX3 (**3**) (Figure 5B), suggesting that the unknown compound detected in *A. circinalis* (TA04) in Figure 2A is **3**, whereas 12α-deoxyGTX2/3 and 12β-deoxyGTX2 (**5**) were not detected in this cyanobacterium. The peak detected at 44.2 min in *A. circinalis* (TA04) (Figure 5C) was identified as GTX 5 (B1) (**4**) by comparison with the authentic standard (not shown). Other peaks shown in Figure 5C were not identified. Furthermore, we confirmed that the MS/MS spectrum of synthetic **3** (Figure 6) was almost identical to that of the unknown compound found in *A. circinalis* (TA04) (Figure 2C).

## 3. Discussion

**Figure 7:**
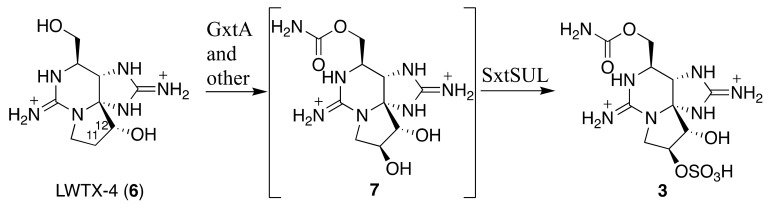
One of the possible biosynthetic routes from **6** to **3**. Compound **7** has not been identified in *A. circinalis* (TA04).

In the present study, 12β-deoxyGTX3 (**3**) was identified in the PST producing cyanobacterium, *A. circinalis* (TA04), whereas its diastereomers at C11 (**5**) and/or at C12 were not detected. In the PST producing dinoflagellate, *Alexandrium tamarense* (Axat-2), 12β-deoxydecarbamoylSTX was previously identified [29] and the same compound was detected in *L. wollei* (LWTX-4) (**6**, Figure 1 and Figure 7) [5,19]. Lim et al. [30] also reported the presence of 12-deoxyGTX4 in *Alexandrium minutum*, although the stereochemistry at C12 of this compound has not been identified. The 12β-deoxyGTX3 (**3**) found in the present study has not been identified in *A. tamarense* (Axat-2). In *L. wollei*, two more 12β-deoxy type STX analogues (LWTX-1 (**2**) and LWTX-5, Figure 1) have been reported [19], whereas 12α-deoxy type analogues have not been previously identified from any natural sources. Mihali et al. [31] reported that a dioxygenase, *sxtdiox,* is a unique gene to the PST geneclusters of *L. wollei* and *Raphidiopsis brookii* D9, and they proposed that *sxtdiox* carries out the hydroxylation at C12 in the biosynthesis of 12-deoxy type PST analogues. However, in a recent report, *sxtDIOX* was predicted to be involved in C11-hydroxylation [32]. Narayan’s group recently characterized the substrate specificities of SxtT and GxtA, both Rieske oxygenases, which catalyze 12α-hydroxylation and 11β-hydroxylation, respectively [16]. Therefore, this stereoselectivity might be a reason to explain why only 12β-deoxy type STX analogues are present in nature. In addition, Narayan’s group characterized the *O*-sulfotransferase for 11β-OH (SxtSUL) [17]. If GxtA and SxtSUL are involved in the production of 12β-deoxyGTX3 (**3**) in *A. circinalis* (TA04), **3** is predicted to be biosynthetically derived from LWTX-4 (**6**) via **7** (Figure 7), although **7** has not been identified in natural sources. Identification of **3** in the present study supports these stereoselective enzymatic oxidation at C11 and C12, and C11-*O*-sulfation, during the late stage of PST biosynthesis.

## 4. Methods and Materials

### 4.1. General Information

The reagents were purchased from Sigma–Aldrich Co. (St. Louis, MO, USA), Wako Pure Chemical Industries, Ltd., Tokyo Chemical Industry Co., Ltd. (Osaka, Japan), and Nacalai Tesque, Inc. (Kyoto, Japan). LC/MS-grade acetonitrile (Wako Pure Chemical Industries, Ltd.) was used for HR–LC–MS. Distilled and purified water (MilliQ) by Simplicity UV (Merck Millipore Corporation, Billerica, MA, USA) was used for all experiments. NMR spectra were recorded at 20 °C with an Agilent 600 MHz NMR spectrometer (Agilent Technologies, Inc., Santa Clara, CA, USA) with CD_3_COOD–D_2_O (4:96, *v*/*v*). Spectra were referenced to CHD_2_COOD signals with resonances at δ_H_ = 2.06 ppm. HR–LC-MS was performed with a micrOTOF-Q II (ESI, Q-TOF) (Bruker Daltonics Inc, Billerica, MA, USA), and column switching LC–MS/MS was performed with an API2000 (AB Sciex, Foster City, CA, USA).

### 4.2. Preparation of 12β-deoxyGTX2 (**5**) and 12β-deoxyGTX3 (**3**) from C1/C2

The mixture of C1 and C2 (C1/C2, 980 µg by LC–MS) was purified from the culture of *A. circinalis* (TA04) (2 L) by chromatography on activated charcoal (for chromatography, 63–300 µm >40%, Wako Pure Chemical Industries), Bio-gel P2, and Macroprep CM (BioRad) and subsequently hydrolyzed to GTX2/3 (153 µg after purification with activated charcoal for chromatography and Hitachigel #3013-C) by heating at 100 °C for 15 min in 0.13 M HCl as reported by Watanabe et al. [26] Next, the aqueous solution of the purified GTX2/3 was neutralized at pH 7–8 with 2.5 M NH_4_OH aqueous and filled with water to a final volume of 0.2 mL. The resulting solution was transferred to another microtube containing 5 mg of NaBH_4_ powder, which was placed on ice. The mixture was vortexed and kept at 0 °C for 30 min. After the reaction, the solution was acidified with 2 mL of 0.5 M AcOH and washed with 2 mL of EtOAc. The water layer was concentrated and neutralized with 1 M NaOH aqueous, and subsequently applied to an activated charcoal column (0.3 mL, for chromatography). The mixture was eluted from the activated charcoal column with 1.5 mL of AcOH-EtOH-H_2_O 5:45:50 (*v*/*v*/*v*) after washing with 0.9 mL of water. The produced 12β-deoxyGTX2/3, with trace amounts of 12α-deoxyGTX2/3, was confirmed by LC–MS analysis. 12β-deoxyGTX3 (**3**) and 12β-deoxyGTX2 (**5**) (approximately 50 µg and 75 µg, respectively, estimated by ^1^H NMR) were obtained after HPLC purification with an InertSustain AQ-C18 (0.46 × 25 cm) column with 0.1% formic acid in water as a mobile phase; compound **3** was eluted earlier than **5**. Purity was confirmed by ^1^H NMR. The yields of almost pure **3** and **5** from C1/C2 were approximately 7% and 10% (mol/mol), respectively.

### 4.3. Preparation of the Mixture of 12α-deoxyGTX2/3 from GTX2/3

The mixture of GTX2/3 (2 µg), similarly prepared from C1/C2 as described above, dissolved in MeOH (0.3 mL) was reacted with NaBH_4_ (2 mg) at 55–60 °C for 75 min [27]. After the reaction was quenched by an addition of 0.5 M AcOH (1.5 mL), the mixture was washed with EtOAc (1.5 mL). The produced mixture of 12α-deoxyGTX2/3 in the water layer was purified using activated charcoal (0.1 mL, for chromatography) as described above for LC–MS analysis. Production of 12α-deoxyGTX2/3 was suggested by HR–LC–MS retention times which were different from those of 12β-deoxyGTX2/3 (**5**, **3**, see, Figure 5A). HRMS [M + H]^+^ *m*/*z* 380.0999 C_10_H_18_N_7_O_7_S, ∆ 3.2 ppm (the peak at 24.9 min, Figure 5A) and *m*/*z* 380.1022 C_10_H_18_N_7_O_7_S, ∆ 4.9 ppm (the peak at 27.0 min, Figure 5A).

### 4.4. Harvest and Preparation of A. circinalis (TA04) Cell Extract for Screening

The toxic strain of the freshwater cyanobacterium *A. circinalis* used in this study is a nonaxenic strain TA04. The field sample of *A. circinalis* was collected at the Tullaroop reservoir, Victoria, Australia, and the TA04 strain was one of single-trichome isolates prepared by Negri et al. [33]. *A. circinalis* (TA04) was provided by Dr. Susan Blackburn, CSIRO, Australia, and cultured in CB’ medium [see, ref.12, SI] (30 mL). The cells were harvested by filtration using a glass fiber filter (GA100, 1.0 μm, Advantec, Tokyo, Japan), suspended with 2.0 mL of 0.5 M AcOH and sonicated three times for 30 s on ice. Then, the resulting solutions were centrifuged at 20,000× *g* for 5 min at 4 °C. The supernatants were filtered through a Cosmospin filter H (0.45 μm, Nacalai Tesque, Kyoto, Japan). A part (200 μL) of the filtrate was adjusted to pH 7–8 using 2 M NH_3_ aqueous, and loaded on an activated charcoal column (100 μL vol.) (for chromatography, 63–300 µm >40%, Wako Pure Chemical Industries, Ltd.). After the column was washed with water (0.2 mL), PST were eluted with AcOH/EtOH/H_2_O (5:50:45, *v*/*v*/*v*, 1 mL). The solvent was removed using N_2_ gas, and the resulting residue was resuspended with 100 μL of 0.05 M AcOH.

### 4.5. HR–HILIC–LC–MS Conditions for the Screening for Novel PST

For the screening for novel PST, HR–hydrophilic interaction liquid chromatography (HILIC) LC–MS was performed on a TSKgel Amide-80 column (2.0 i.d. × 150 mm, 5 μm, Tosoh, Tokyo, Japan). The mobile phase was 2 mM HCOONH_4_ buffer containing CH_3_CN/water (62:38, *v*/*v*) [24]. The flow rate was 0.2 mL min^−1^. The oven temperature was 25 °C.

### 4.6. MS Conditions for HR–LC–MS and HR–LC–MS/MS

HR–LC–MS were recorded on a micrOTOF-Q II mass spectrometer (Bruker Daltonics Inc. Billerica, MA, USA) equipped with an ESI ion source. The liquid chromatography system used for analysis was a Shimadzu Nexera UHPLC System (Shimadzu, Kyoto, Japan). The mass spectrometer conditions were as follows: Positive ionization mode; dry gas: nitrogen 7 L min^−1^; dry heater temperature: 180 °C; nebulizer: 1.6 Bar; capillary: 4500 V. HR–LC–MS/MS was performed in AutoMS/MS mode setting [M + H]^+^ as the precursor ions. The precursor ions were *m*/*z* 380.10 for 12β-deoxyGTX3 (**3**) and GTX5 (**4**) width 3 Da. The sweeping collision energy was 41–62 eV.

### 4.7. Column-Switching LC–MS/MS (MRM) Conditions [28]

For the analysis of 11,12-diastereomers of 12-deoxyGTX2/3, the column-switching LC-MS method was utilized on three guard cartridge columns: Develosil C30-UG (4.0 i.d. × 10 mm, Nomura Chemical, Seto, Japan), TSKgel Guardgel Amide-80 (5 µm, 2.0 i.d. × 10 mm, Tosoh, Tokyo, Japan), and SeQuant ZIC-HILIC Guard (2.1 i.d. × 20 mm, Merck KGaA, Darmstadt, Germany), as extraction columns and a SeQuant ZIC-HILIC metal-free HPLC column (PEEK, 2.1 i.d. × 150 mm, 5 µm, Merck KGaA, Billerica, MA, USA) as an analytical column. Mobile phase A was 200 mM HCOONH_4_ buffer containing 200 mM HCOOH/water (5.0:95, *v*/*v*, pH 3.9), and mobile phase B was 200 mM HCOONH_4_ buffer containing 200 mM HCOOH/water/CH_3_CN (5:1.5:95, *v*/*v*/*v*). A gradient elution program was applied as follows: Initial: 100% B, 0–3.1 min: 100 % B, 3.1–6 min: 85% B, 6–14 min: 85%–70% B, 14–16 min: 70% B, 16.1–30 min: 85% B, 30.1–35 min: 45% B, 35.1–49.9 min: 85% B, 50–51 min: 40% B, 51.1–62 min: 100% B. The flow rate was initially set at 0.2 mL min^−1^, changed gradually to 0.3 mL min^−1^ (0–3.05 min), and then kept at 0.3 mL min^−1^ (3.05–62 min). The position of the Valco valve was set to ‘A’ (to waste) from 0 to 2 min, ‘B’ (to column) from 2 to 50 min, and ‘A’ (to waste) from 50 to 62 min. An API2000 triple quadrupole tandem mass spectrometer (AB Sciex, Framingham, MA, USA) equipped with an ESI ion source was utilized. LC was performed using two HPLC pumps (LC-10AD, Shimadzu, Kyoto, Japan). The mass spectrometer conditions were as follows: Positive ionization mode; dry gas: nitrogen; spray voltage: 4.5 kV; capillary temperature: 500 °C; curtain gas: 40 (arbitrary units), GS1: 60; GS2: 80; ihe: ON; CAD: 6. In the multiple reaction monitoring (MRM), the precursor ions to the product ions (Q1/Q3) and collision energy (CE in eV) were chosen as *m*/*z* 380.1/300.0 (31). The parameters for the mass spectrometer were set to DP (21), FP (400), EP (10), CEP (18), and CXP (4).

## Figures and Tables

**Table 1:** ^1^H NMR (600 MHz) data for synthetic 12β-deoxyGTX2 (**5**) and 12β-deoxyGTX3 (**3**), and LWTX-1 (**2**) [19].

	12β-deoxyGTX2 (5)		12β-deoxyGTX3 (3)		LWTX-1 (2)	
No.	δ_H_	Multiplicity (*J* in Hz)	δ_H_	Multiplicity (*J* in Hz)	δ_H_	(*J* in Hz) ***
5	4.74	s	4.77	s	4.74	s
6	3.90	t 4.3	3.90	t 4.3	3.93	4.8
10α	4.17	d 12.3	4.15	dd 11.5, 11.9	4.16	8.1, 11.6
10β	3.96	dd 11.5, 4.4	3.72	dd 11.5, 4.3	3.67	5.4, 11.6
11	5.07	t 3.1	4.91 *	n.d. **	4.90	5.4, 7.2, 8.1
12	4.45	d 4.0	4.52	d 7.0	4.49	7.2
13a	3.96	m	4.27	dd 13.9, 5.6	4.29	4.8
13b	4.29	dd 11.5, 4.4	4.33	dd 13.9, 4.4	4.29	4.8

CD_3_COOD-D_2_O (4:96, *v*/*v*). The signal of CHD_2_COOD (2.06 ppm) was used as an internal reference. * estimated by TOCSY correlation, ** not determined, *** multiplicity is not shown in the reported data [19].

## Supplementary Materials

The following are available online at https://www.mdpi.com/2072-6651/11/9/539/s1, Figure S1: ESI–HRMS spectrum of synthetic 12β-deoxyGTX3 (**3**), Figure S2: ESI–HRMS spectrum of synthetic 12β-deoxyGTX2 (**5**), Figure S3: Separation of **3** and **5** using reverse-phase chromatography, Figure S4: COSY spectrum of synthetic **3**, Figure S5: TOCSY spectrum of synthetic **3**, Figure S6: COSY spectrum of synthetic **5**, Figure S7: TOCSY spectrum of synthetic **5**.
