# Supplementary material for: Identification of a Novel Saxitoxin Analogue, 12β-Deoxygonyautoxin 3, in the Cyanobacterium, Anabaena circinalis (TA04)
Source: Toxins (Basel). 2019 Sep 16;11(9):539. doi: 10.3390/toxins11090539 (PMC6784053; doi:10.3390/toxins11090539)
Supplement: Supplementary file 1 [file toxins-11-00539-s001.pdf]

Supplementary Materials: Identification of a Novel Saxitoxin Analogue, 12β-Deoxygonyautoxin 3, in the Cyanobacterium, *Anabaena circinalis* (TA04)

Takashi Minowa, Yuko Cho, Yasukatsu Oshima, Keiichi Konoki and Mari Yotsu-Yamashita

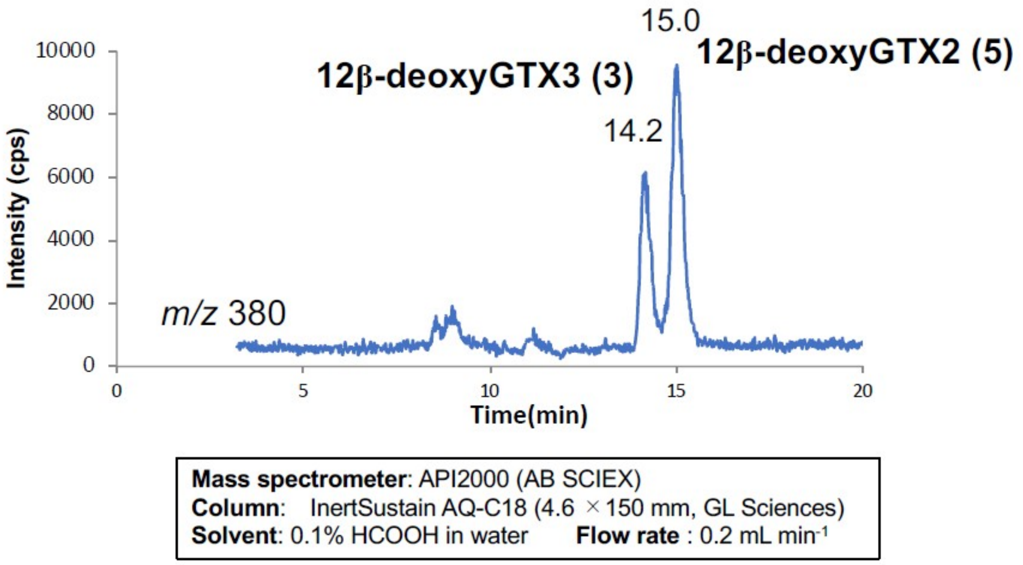

Figure S1. Separation of **3** and **5** using reverse-phase chromatography.

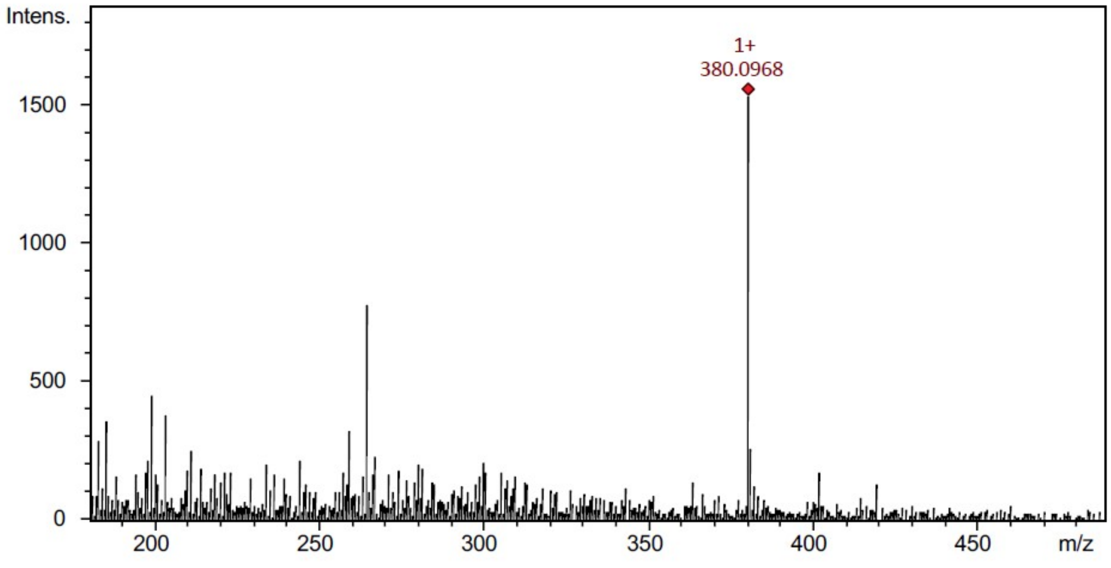

Figure S2. ESI-HRMS spectrum of synthetic 12β-deoxyGTX3 (**3**) ($[M+H]^+$, *m*/*z* 380.0968 C10H18N7O7S, Δ 3.9 ppm).

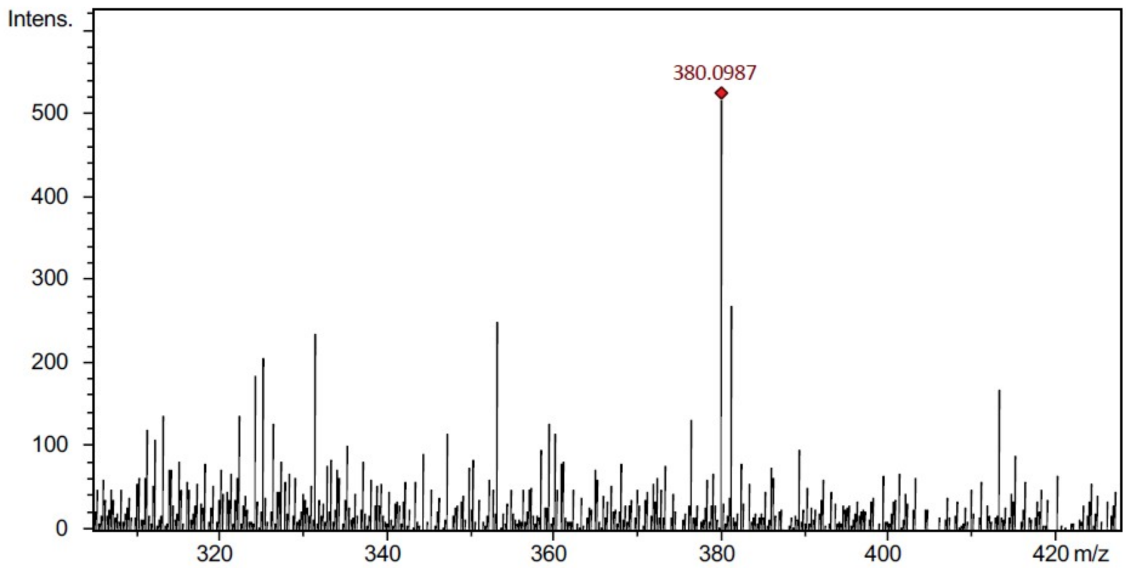

Figure S3. ESI-HRMS spectrum of synthetic 12β-deoxyGTX2 (**5**). ([M+H]+, *m*/*z* 380.0987 C10H18N7O7S, Δ 1.1 ppm)

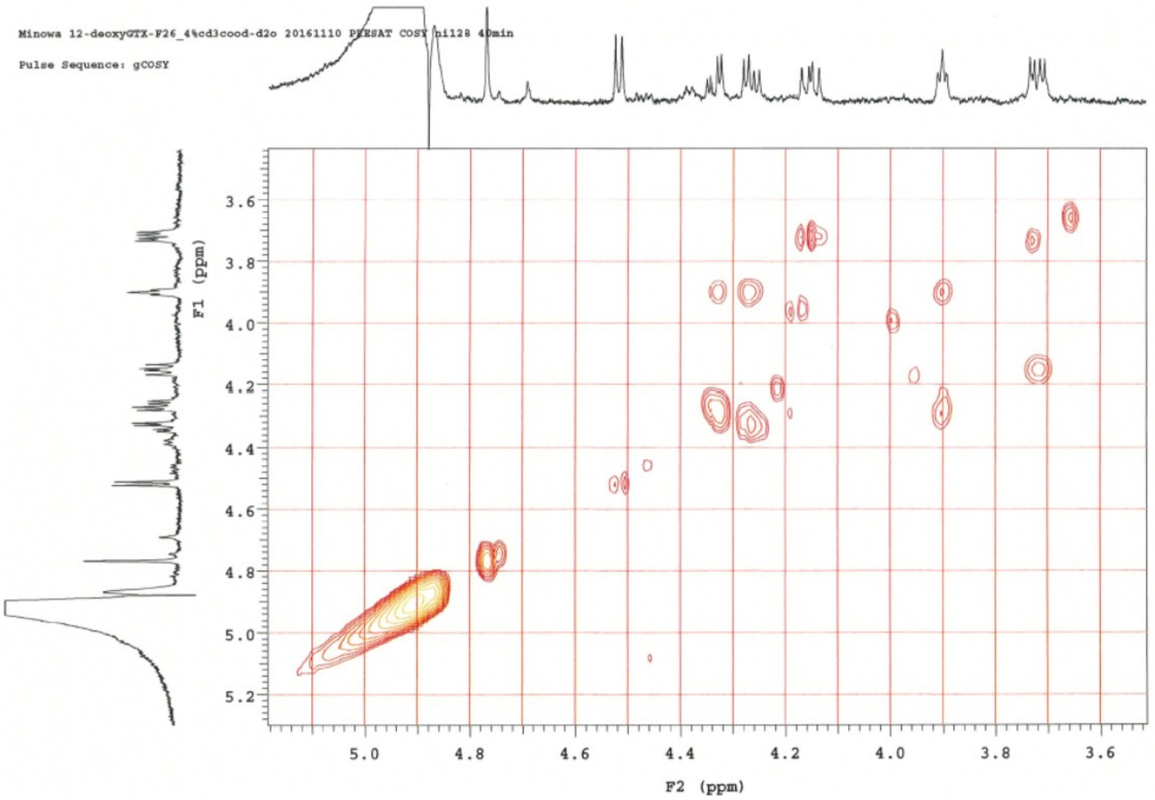

Figure S4. COSY spectrum of synthetic 12β-deoxyGTX3 (**3**),.600 MHz, CD3COOD-D2O (4:96, *v*/*v*).

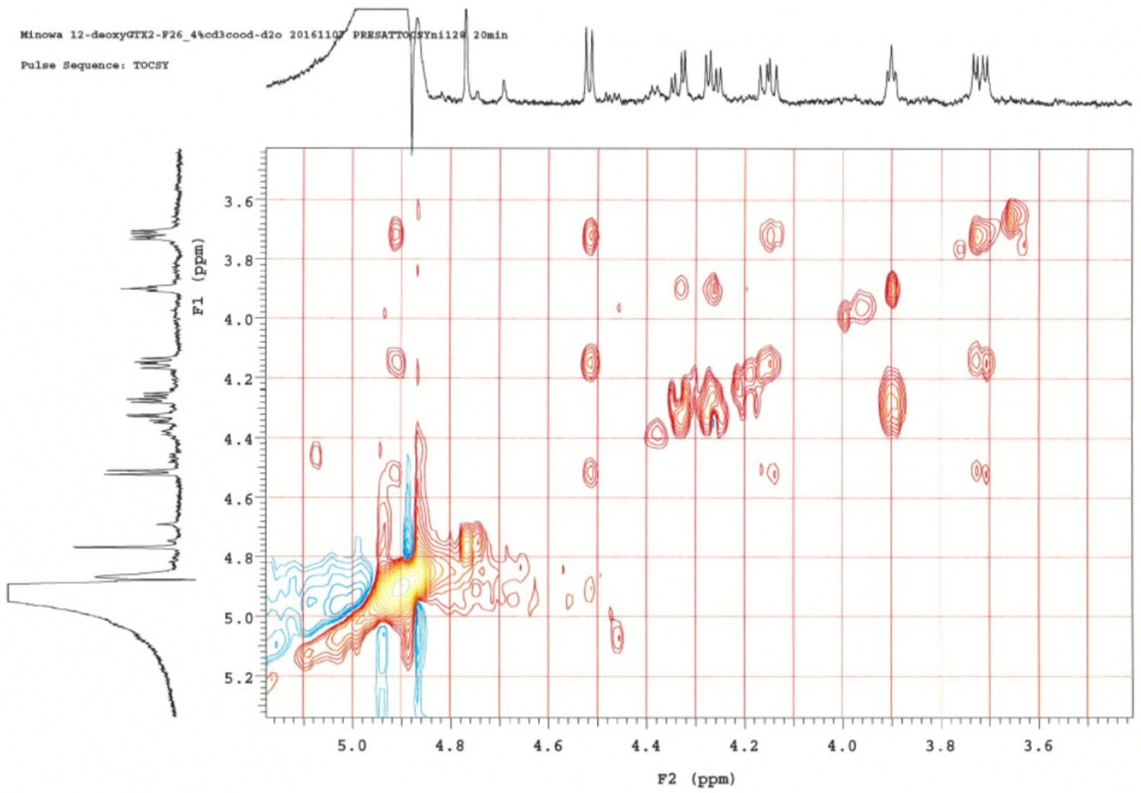

Figure S5. TOCSY spectrum of synthetic 12β-deoxyGTX3 (**3**), 600 MHz, CD3COOD-D2O (4:96, *v*/*v*)

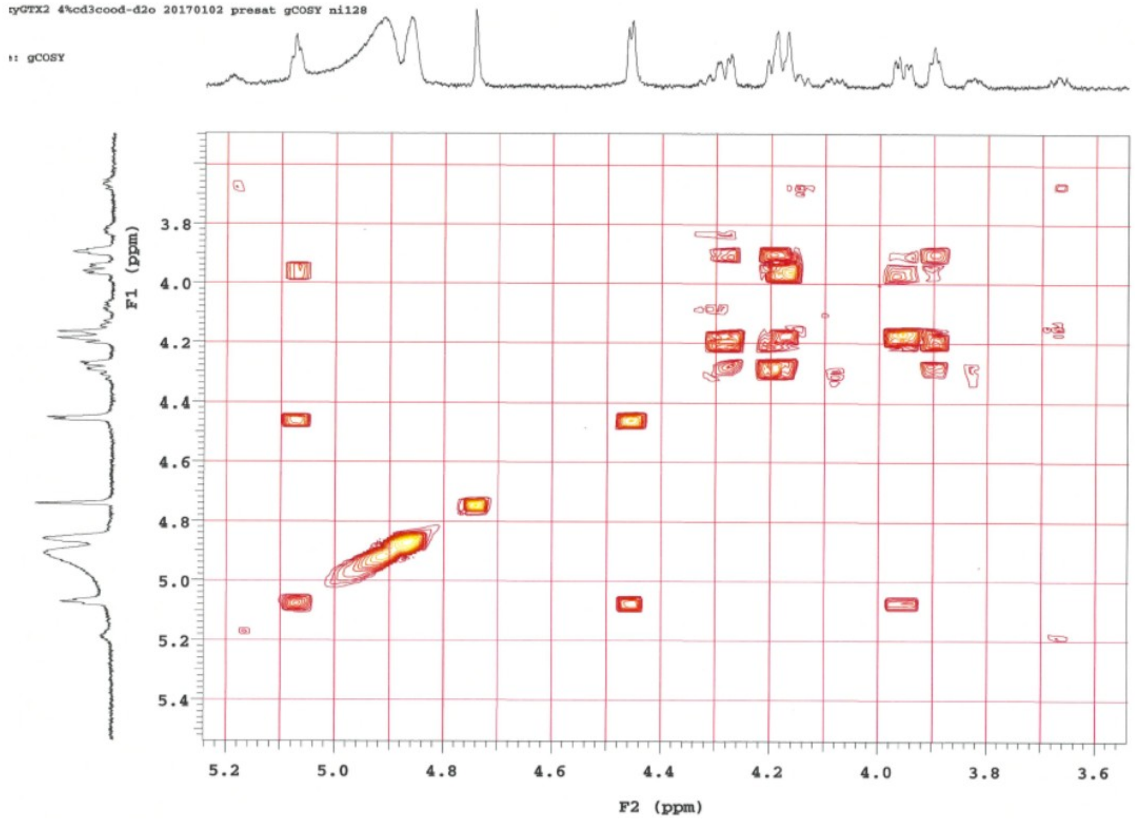

Figure S6. COSY spectrum of synthetic 12β-deoxyGTX2 (**5**), 600 MHz, CD3COOD-D2O (4:96, *v*/*v*).

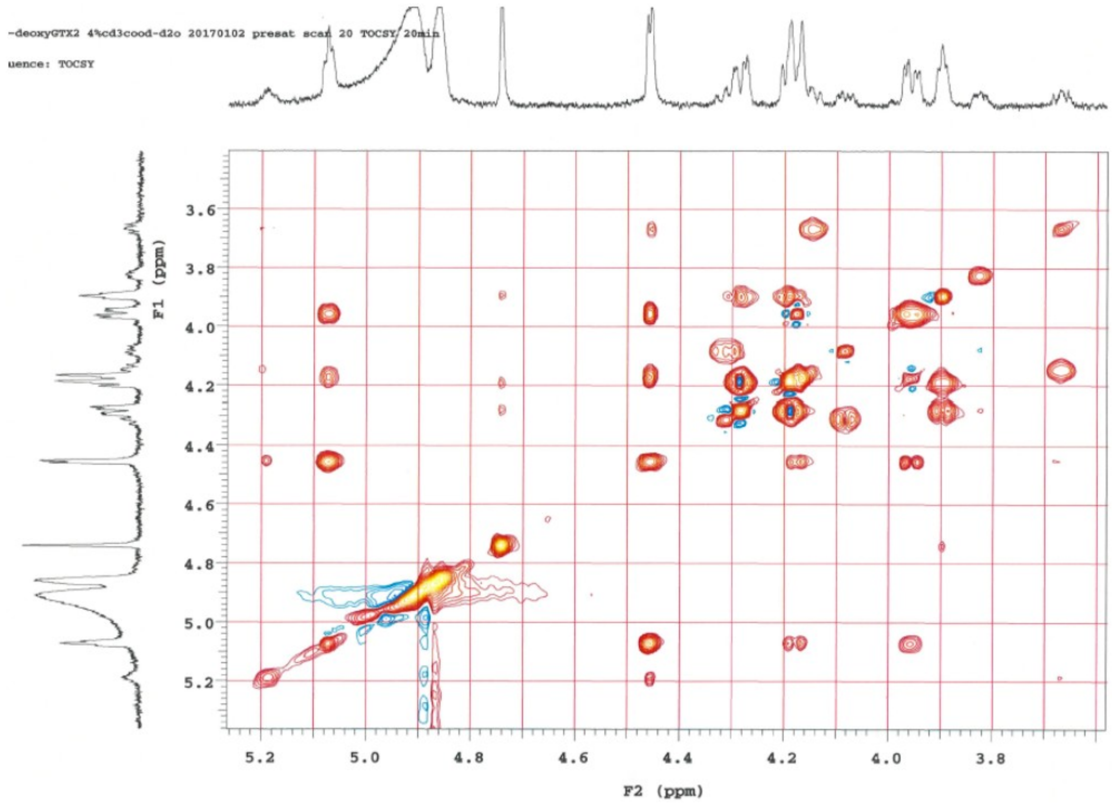

Figure S7. TOCSY spectrum of synthetic 12β-deoxyGTX2 (**5**), 600 MHz, CD_3COOD-D_2O (4:96, *v*/*v*).
